# Molecular Assessment of *Scutellaria barbata* D. Don in the Treatment of Nasopharyngeal Carcinoma Based on Network Pharmacology and Experimental Verification

**DOI:** 10.1155/2022/1988378

**Published:** 2022-02-07

**Authors:** Hongjian Shi, Jie Liu, Jingying Fan, Lan He, Xianwen Wang, Faqing Tang, Daofa Tian, Yingchun He

**Affiliations:** ^1^Hunan University of Chinese Medicine, Changsha 410208, China; ^2^Hunan Provincial Key Laboratory for Prevention and Treatment of Ophthalmology and Otolaryngology Diseases with Chinese Medicine, Changsha 410208, China; ^3^Hunan Provincial Engineering and Technological Research Center for Prevention and Treatment of Ophthalmology and Otolaryngology Diseases with Chinese Medicine and Protecting Visual Function, Changsha 410208, China; ^4^The first hospital of Hunan University of Chinese Medicine, Changsha 410208, China

## Abstract

**Objective:**

To predict the molecular mechanisms behind the benefits of *Scutellaria barbata* D. Don (*S. barbata*) in nasopharyngeal carcinoma (NPC) by network pharmacology and experimental verification.

**Methods:**

The active ingredients and targets of *S. barbata* were searched in the traditional Chinese medicine system pharmacology database and analysis platform, and the disease targets of NPC were obtained by searching the GeneCards database. A common target protein-protein interaction network was constructed by STRING, and then, an active ingredients-NPC-target interaction network map was constructed by Cytoscape 3.7.2 software. The functional enrichment analyses of Gene Ontology and KEGG pathway data were carried out by R software programming. Finally, cell proliferation was assessed by CCK8, apoptosis was detected by Annexin V-FITC/PI double fluorescence staining, and protein expression was analyzed by Western blotting.

**Results:**

In this study, 29 active ingredients were found in *S. barbata*. Among these, the main targets for NPC were baicalein, wogonin, luteolin, and quercetin. The main molecular targets of *S. barbata* on NPC were EGFR, MYC, CASP3, CCND1, and ESR1. The main biological processes involved the binding of DNA-binding transcription factors, RNA polymerase II-specific DNA-binding transcription factors, ubiquitin-like protein ligases, and ubiquitin-protein ligases. *S. barbata* mainly affects NPC through the PI3K-Akt, p53, and MAPK signaling pathways. The experimental results showed that baicalein and wogonin could inhibit proliferation and induce apoptosis of NPC cells and downregulate the expression of PI3K, AKT, and p53, the key proteins of the PI3K/AKT and p53 signaling pathway in CNE2 cells.

**Conclusion:**

Baicalein and wogonin, the main active ingredients of *S. barbata*, inhibited the proliferation and induced apoptosis of NPC cells through the PI3K/AKT and p53 signaling pathways.

## 1. Introduction

Nasopharyngeal carcinoma (NPC) is a malignant tumor of the head and neck, which originates from the epithelial tissue of nasopharyngeal mucosa. According to the data of the International Agency for Research on Cancer, 129079 cases of NPC were added in 2018, and 72987 patients died [1]. NPC is sensitive to radiotherapy. However, due to its concealed location, it is difficult to distinguish symptoms from benign diseases. Most NPC patients are at middle and late stages when they are diagnosed and accompanied by cervical lymph nodes and/or distant metastasis, resulting in a high rate of treatment failure [2]. At present, the main treatment for NPC is radiotherapy combined with platinum-based chemotherapy. However, conventional chemotherapy drugs have great toxicity and side effects, and a single drug is prone to drug resistance. A large number of studies have shown that traditional Chinese medicine (TCM) can effectively improve the efficacy of chemotherapy, radiotherapy, targeted therapy, and immunotherapy [3]. Wang et al. retrospectively evaluated the Taiwan Cancer Registry database for patients with advanced NPC from 2007 to 2013 and found that the overall risk of death in patients using Chinese herbal medicine was 0.799 times the control group, and *Scutellaria barbata* D. Don (*S. barbata*) was found to be an effective TCM for treating NPC [4].

*S. barbata* is a TCM, belonging to the genus *Scutellaria* of the Labiatae family. Modern pharmacological studies show that *S. barbata* extract has many pharmacological activities, such as antimicrobial, anti-inflammatory, and antitumor [5]. The use of *S. barbata* in antitumor treatment and research has received more and more attention, especially for colon, breast, liver, and pancreatic cancers [6–8]. The results of clinical treatment and experimental studies show that the pharmacological effects of *S. barbata* mainly include inhibiting the proliferation, migration, and invasion of tumor cells, antitumor angiogenesis, and promoting apoptosis [9–11]. However, the material basis and mechanism of *S. barbata* in the treatment of NPC remain unclear.

Network pharmacology is a new strategy of multitarget drug molecular design by selecting specific signal nodes through the network analysis of biological systems in a network database and based on the theory of systems biology. In recent years, network pharmacology has been widely used in the research of TCM to provide a new tool and method for exploring their actions of mechanism and developing active ingredients [12]. This study combines network pharmacology with experimental verification to clarify the characteristics and potential molecular mechanism of *S. barbata* in the treatment of NPC and to provide a reference for the development of TCM and monomers to inhibit NPC.

## 2. Materials and Methods

### 2.1. Screening Active Ingredients of *S. barbata*

*S. barbata* was searched in the traditional Chinese medicine system pharmacology database and analysis platform (TCMSP) database (https://old.tcmsp-e.com/index.php). The active ingredients and action targets of *S. barbata* were obtained by screening oral bioavailability (OB) ≥ 30% and drug-like (DL) ≥ 0.18 substances.

### 2.2. Screening of Disease Targets for NPC

The keyword “nasopharyngeal carcinoma” was searched through the GeneCards database (https://www.genecards.org/) to obtain the disease target of NPC.

### 2.3. Screening Common Targets of Active Ingredients and Diseases and Construction of the Protein-Protein Interaction Network

The disease target was intersected with the drug target by R software (https://www.r-project.org/), and a Venn diagram was drawn. The protein-protein interaction (PPI) network was constructed with STRING plug (https://string-db.org/). The protein type was set to “*Homo sapiens*,” and other parameters were set to default to obtain the PPI network. The frequency of common protein targets was obtained by R software.

### 2.4. Drug Active Ingredients-NPC-Target Interaction Network

The interaction network between the active ingredients of *S. barbata* and disease targets was constructed by Cytoscape 3.7.2 software, and an interaction network diagram of drug active ingredients-NPC-targets was drawn to explore the potential mechanism of *S. barbata* in the treatment of NPC.

### 2.5. Biological Function Analysis of Gene Ontology (GO) and KEGG Pathway Enrichment Analysis

The common target sites of *S. barbata* active ingredients and NPC were analyzed by GO and KEGG pathway enrichment analysis with R software (https://www.r-project.org/). GO analysis was mainly used to describe the functions of gene products, including cellular, molecular, and biological functions. KEGG pathway enrichment analysis was used to analyze core pathways and explore the possible biological function and signaling pathway mechanisms in the treatment of NPC.

### 2.6. Experimental Verification

NPC CNE2 and 5-8F cells were treated with different concentrations of baicalein and wogonin, respectively.. The cell proliferation was assessed by the CCK8 method. The apoptosis rate was determined by Annexin V-FITC/PI double fluorescence staining, and the expression level of key proteins of the PI3K/AKT and p53 signaling pathway was analyzed by Western blotting.

### 2.7. Statistical Analysis

The data were processed by SPSS 26.0 statistical software and presented as the mean ± standard deviation if they obeyed normal distribution. One way ANOVA was used to compare the measurement data between single-factor design and multiple groups. The least significant difference method was used for multiple comparisons, and the Games–Howell test was used to test the variance. The results analysis map was made by GraphPad Prism 8.0 software.

## 3. Results

### 3.1. Screening the Active Ingredients of *S. barbata*

The active ingredients of *S. barbata* were searched in the TCMSP database, and a total of 29 active ingredients were obtained. The results are given in Table 1.

### 3.2. Screening of Disease Targets for NPC

After searching the GeneCards database and setting a relevance score at ≥5 for preliminary screening, 722 therapeutic targets of NPC were obtained.

### 3.3. Common Target Screening and Interactive Network Construction

**Figure 1:**
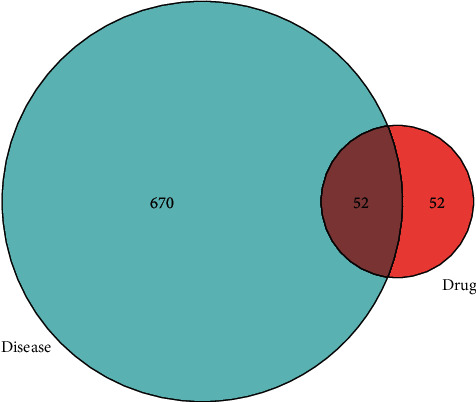
Venn diagram of the action targets of active ingredients of *S. barbata* and the therapeutic targets of NPC.

**Figure 2:**
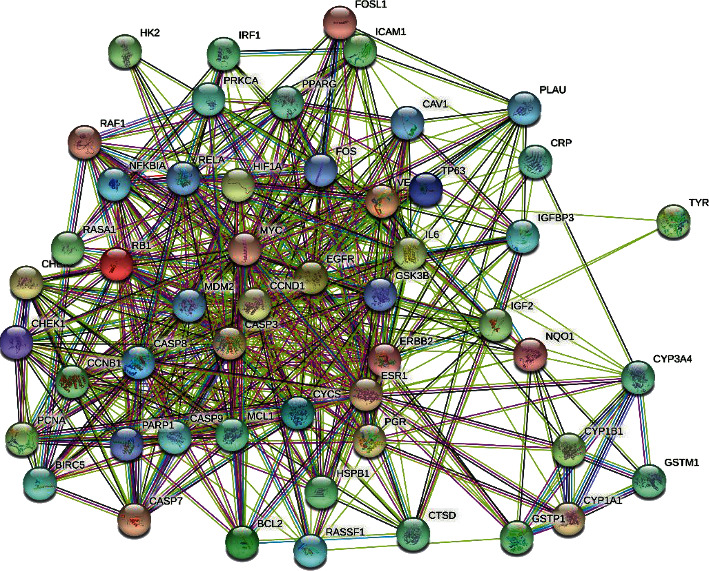
The protein-protein interaction (PPI) network.

**Figure 3:**
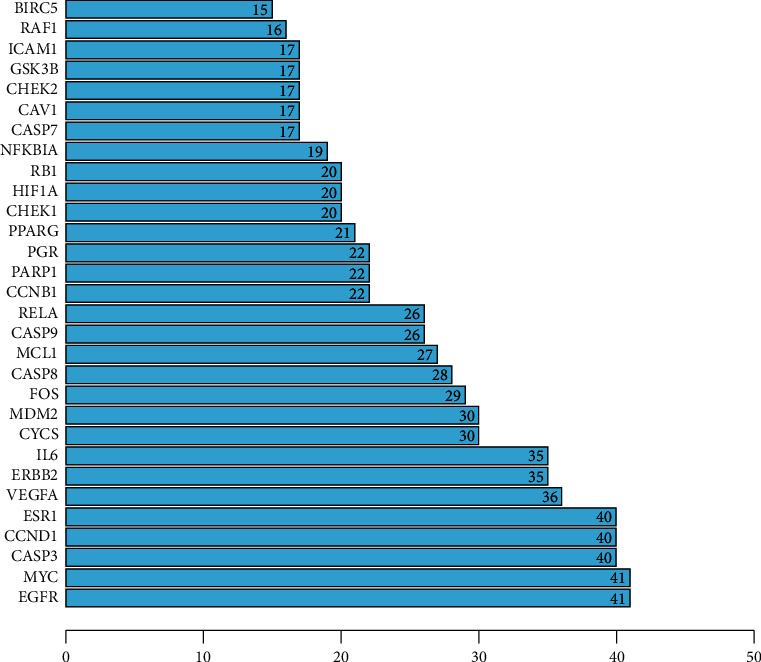
Frequency of common protein targets.

The therapeutic targets of NPC and the action targets of the active ingredients of *S. barbata* were screened by R software, and 52 common targets were obtained (Figure 1). The 52 common targets were entered into the STRING data platform to build the PPI network (Figure 2). The frequency of the occurrence of each protein target was calculated by R software. The most frequent protein targets were EGFR, MYC, CASP3, CCND1, and ESR1, and these could be the main potential targets for *S. barbata* in the treatment of NPC (Figure 3).

### 3.4. Construction of a Drug Active Ingredients-NPC-Target Interaction Network

**Figure 4:**
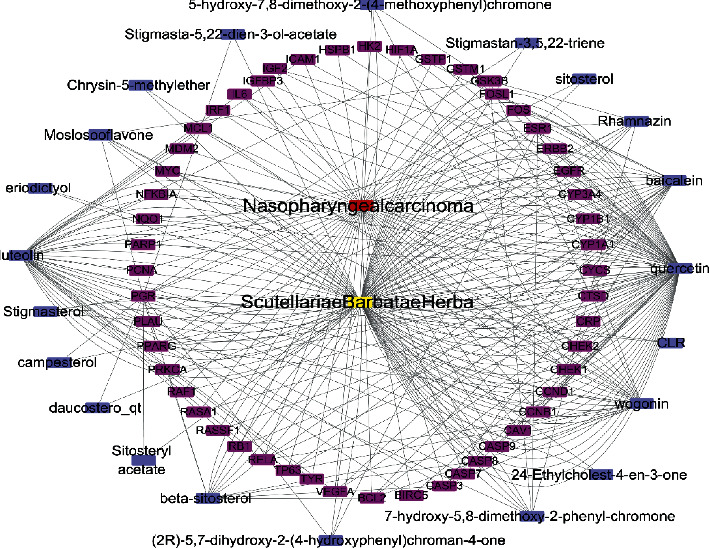
Active ingredients-NPC-target interaction network.

The network diagram of the drug active ingredients-NPC-target interaction network (Figure 4) was drawn by Cytoscape 3.7.2 software. Yellow represents the drug *S. barbata*, red represents the disease NPC, purple represents the active ingredients of *S. barbata*, and rose-red represents the common target of the active ingredients of the drug and NPC. The results indicate that the main active ingredients of *S. barbata* on the common target were baicalein, wogonin, luteolin, and quercetin.

### 3.5. GO Analysis and Core Pathway Screening of *S. barbata* in the Treatment of NPC

**Figure 5:**
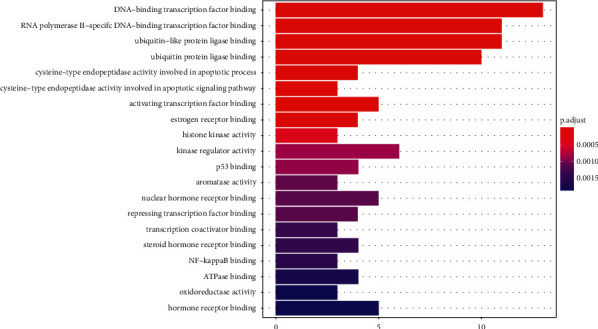
GO analysis bar chart.

**Figure 6:**
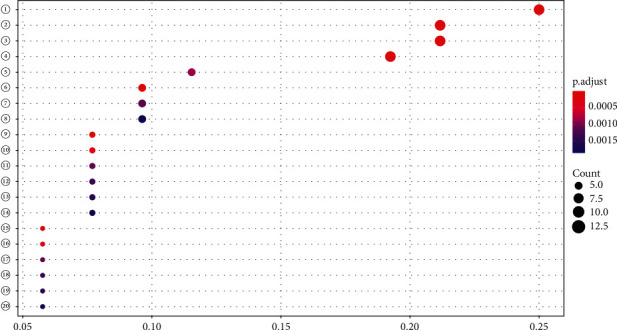
GO analysis bubble chart. The node size represents the number of enrichment targets; the color of the node from red to dark blue indicates that the *P* value is small to large, so the larger the red node, the more significant the signaling pathway, indicating that the signaling pathway is more important. (1) DNA-binding transcription factor binding; (2) RNA polymerase II-specific DNA-binding transcription factor binding; (3) ubiquitin-like protein ligase binding; (4) ubiquitin protein ligase binding; (5) kinase regulator activity; (6) activating transcription factor binding; (7) nuclear hormone receptor binding; (8) hormone receptor binding; (9) cysteine-type endopeptidase activity involved in the apoptotic process; (10) estrogen receptor binding; (11) p53 binding; (12) repressing transcription factor binding; (13) steroid hormone receptor binding; (14) ATPase binding; (15) cysteine-type endopeptidase activity involved in the apoptotic signaling pathway; (16) histone kinase activity; (17) aromatase activity; (18) transcription coactivator binding; (19) NF-kappaB binding; (20) oxidoreductase activity.

The bar chart (Figure 5) and bubble chart (Figure 6) were obtained by GO analysis of the above common targets by R software. It was found that the biological process of *S. barbata* acting on NPC targets mainly involved DNA-binding transcription factor binding, RNA polymerase II-specific DNA-binding transcription factor binding, ubiquitin-like protein ligase binding, and ubiquitin protein ligase binding.

**Figure 7:**
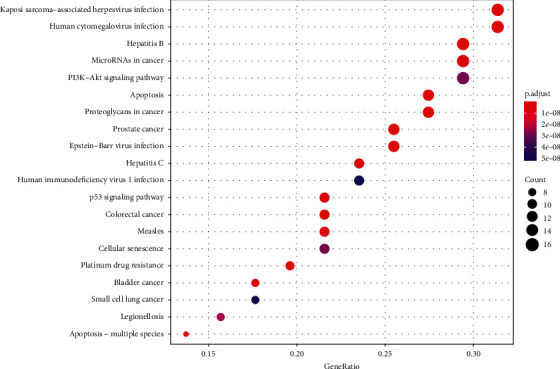
Results of KEGG enrichment analysis.

Figure 7 shows the results of the KEGG pathway enrichment analysis of *S. barbata* acting on NPC targets. The main signaling pathways with the number of enrichment targets ≥10 were PI3K-Akt, MAPK, and p53 signaling pathways (Table 2).

### 3.6. Experimental Results

#### 3.6.1. Baicalein and Wogonin Inhibit the Proliferation of NPC Cells

**Figure 8:**
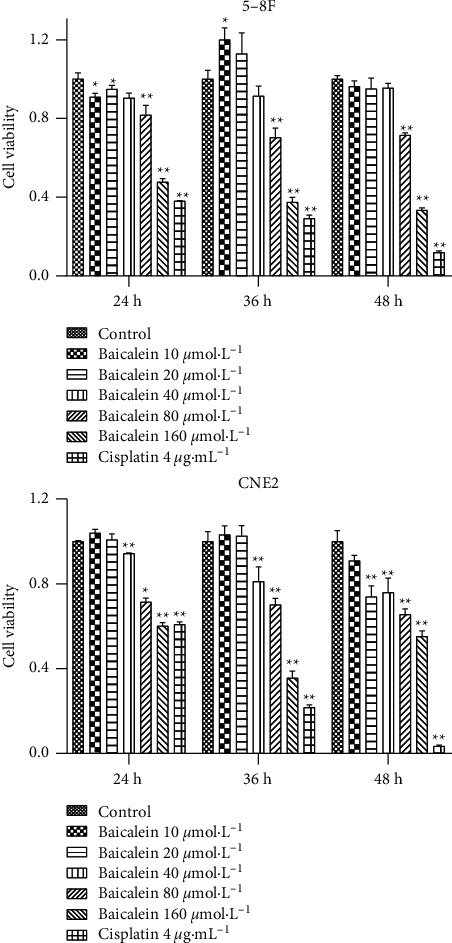
CCK8 assessment of the effect of baicalein on the proliferation of CNE2 and 5-8F cells (vs. the control group: ^*∗*^*P* < 0.05 and ^*∗∗*^*P* < 0.01).

**Figure 9:**
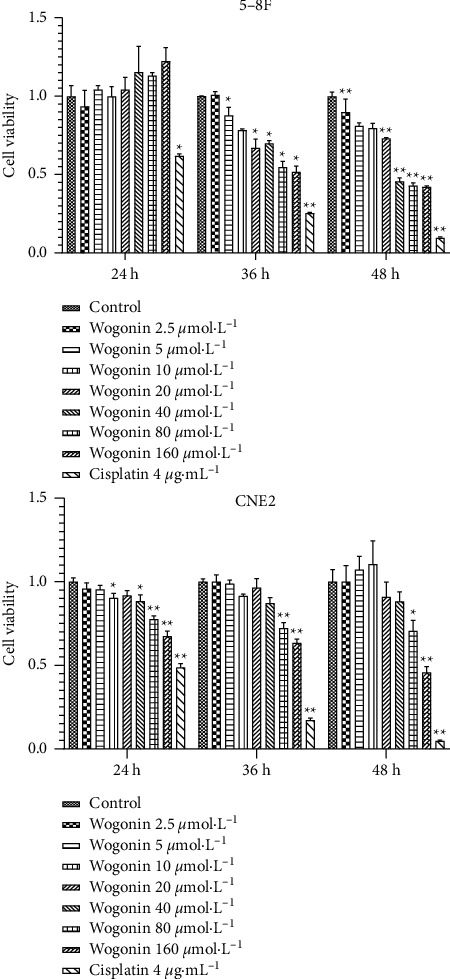
CCK8 assessment of the effect of wogonin on the proliferation of CNE2 and 5-8F cells (vs. the control group: ^*∗*^*P* < 0.05 and ^*∗∗*^*P* < 0.01).

CCK8 results showed that baicalein significantly inhibited the proliferation of CNE2 and 5-8F cells after 24, 36, and 48 hours (Figure 8). Similarly, wogonin can inhibit the proliferation of CNE2 and 5-8F cells (Figure 9).

#### 3.6.2. Baicalein and Wogonin Induce Apoptosis in NPC Cells

**Figure 10:**
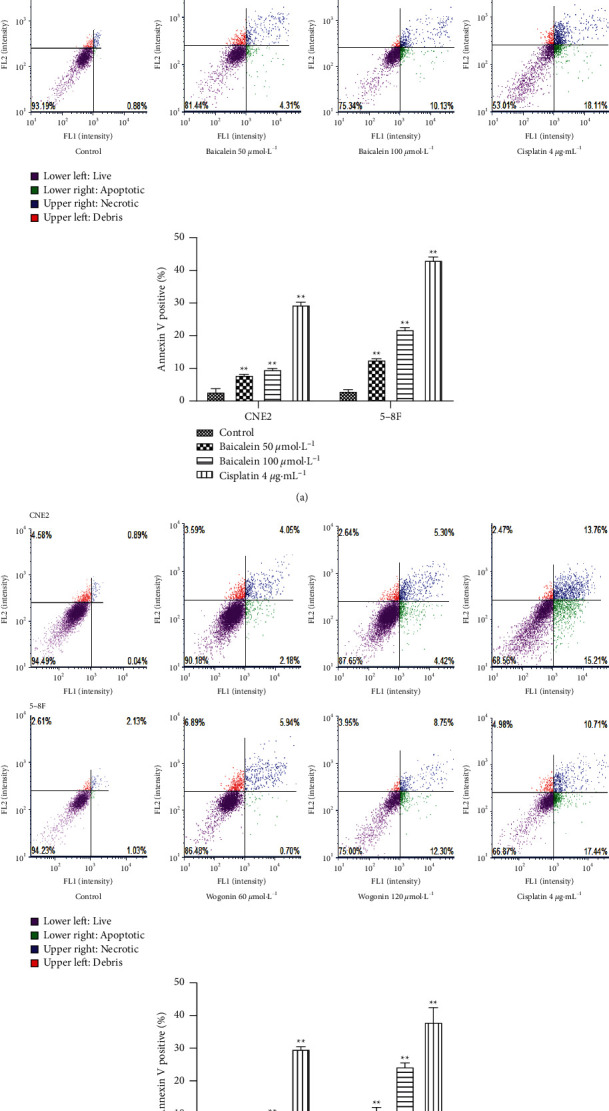
Annexin V-FITC/PI double fluorescence staining used to detect the effect of baicalein and wogonin on apoptosis rate (vs. the control group: ^*∗∗*^*P* < 0.01).

When CNE2 and 5-8F cells were treated with baicalein and wogonin, respectively, the apoptosis rate of CNE2 and 5-8F cells was significantly increased (Figure 10).

#### 3.6.3. Baicalein and Wogonin Inhibit the Activity of Key Proteins in the PI3K/AKT and p53 Signaling Pathway

**Figure 11:**
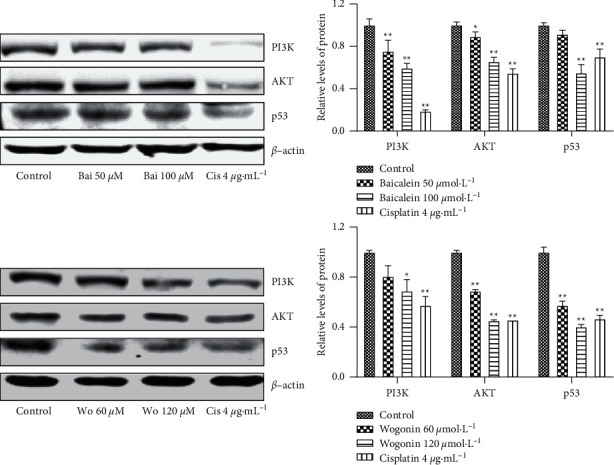
The effects of baicalein and wogonin on the expression of key proteins of the PI3K/AKT and p53 signaling pathway in CNE2 cells analyzed by Western blotting (vs. the control group: ^*∗*^*P* < 0.05 and ^*∗∗*^*P* < 0.01).

As shown in Figure 11, baicalein and wogonin significantly decreased the expression of PI3K, AKT, and p53, the key proteins in the PI3K/AKT and p53 signaling pathway, in CNE2 cells treated with either baicalein or wogonin.

## 4. Discussion

*S. barbata*, also known as “Ban Zhi Lian” in China, has heat-clearing and detoxifying properties (Qingre Jiedu in Chinese) [5]. According to the 2015 edition of the Chinese Pharmacopoeia, *S. barbata* can be used to treat various inflammations, edema, traumatic injury, macula, and snake bites. In recent years, it is often used alone or in combination with other TCM to treat various cancers [13]. For example, Yang et al. found that *S. barbata* combined with *Hedyotis diffusa* can inhibit breast cancer by interfering with the miR-200c-PDE7B/PD-L1-AKT/MAPK axis [14]. Modern pharmacological studies have shown that *S. barbata* contains a variety of active chemical components, including flavonoids, polysaccharides, diterpenes, and porphyrins, which have good antitumor activity and can achieve an antitumor effect through different mechanisms [15].

This study uses network pharmacology to explore the molecular mechanism of *S. barbata* in the treatment of NPC. Through database screening, a total of 29 bioactive ingredients were obtained from *S. barbata*. From the construction of the active ingredients-target interaction network, it was found that the main active ingredients of *S. barbata* were baicalein, wogonin, luteolin, and quercetin. Baicalein and wogonin are flavonoids in *S. barbata*. Related studies have shown that baicalein can exert its antitumor effect by inducing tumor cell apoptosis and invasion, inhibiting tumor angiogenesis and scavenging free radicals [16]. Wogonin can promote the apoptosis of tumor cells, enhance the toxicity of TNF-*α* and TRAIL to tumor cells, block the tumor cell cycle, inhibit tumor angiogenesis, and cooperate with chemotherapeutic drugs through ROS and Ca2+-mediated signaling pathways [17]. We confirmed that both baicalein and wogonin can inhibit the proliferation of NPC CNE2 and 5-8F cells and induce apoptosis by CCK8 and Annexin V-FITC/PI double fluorescence staining, which is consistent with the results of network pharmacological prediction, indicating that the method based on network pharmacology to predict the effective components of TCM is scientific and feasible.

To determine the pharmacological mechanism of *S. barbata* on NPC, we analyzed the active ingredients of *S. barbata* and NPC targets by using a PPI network and found that the protein targets with high frequency were EGFR, MYC, CASP3, CCND1, and ESR1. EGFR is a transmembrane receptor tyrosine kinase that regulates a variety of cellular functions, including cell proliferation, differentiation, and migration [18]. The upregulation of the EGFR signaling pathway is associated with many cancers, including glioblastoma, lung adenocarcinoma, and endometrial carcinoma [19–21]. The expression of MYC is upregulated in NPC and is related to the proliferation and metastasis of NPC cells [22]. The results of GO function analysis showed that the biological processes of *S. barbata* affecting NPC were mainly related to DNA-binding transcription factors, RNA polymerase II-specific DNA-binding transcription factors, ubiquitin-like protein ligase binding, and ubiquitin-protein ligase binding.

KEGG pathway enrichment analysis showed that *S. barbata* affected NPC mainly through the PI3K-Akt, p53, and MAPK signaling pathways. In an acupuncture study of core binding factor-related acute myeloid leukemia, it was found that baicalein could induce apoptosis, accompanied by p53-mediated apoptotic gene expression [23]. Wang et al. found that baicalein inhibits proliferation and induces apoptosis of bladder cancer cells by inhibiting the PI3K/AKT/mTOR signaling pathway in vivo and in vitro [24]. It has been found that wogonin inhibits the proliferation of human colorectal cancer cells by inducing autophagy, apoptosis, and G2/M cell cycle arrest via modulating the PI3K/AKT and STAT3 signaling pathways [25]. Our results also showed that baicalein and wogonin inhibited the expressions of PI3K, AKT, and p53, which are the key proteins in the PI3K/AKT and p53 signaling pathway in CNE2 cells, suggesting that the main active ingredients of *S. barbata* can play an anti-NPC effect through the PI3K/AKT and p53 signaling pathway.

This study systematically predicted the material basis and mechanism of *S. barbata* in the treatment of NPC. Through experiments, it was found that the main active ingredients of *S. barbata*, baicalein and wogonin, could inhibit the proliferation and induce apoptosis of NPC cells and inhibit the activity of the PI3K/AKT and p53 signaling pathway, which provides a theoretical basis for clinical intervention of NPC by *S. barbata* and provides evidence and references for the development of TCM and monomers in the treatment of NPC.

## Figures and Tables

**Table 1:** Main active ingredients of *S. barbata*.

ID	Active ingredients	OB (%)	DL
MOL001040	(2R)-5,7-Dihydroxy-2-(4-hydroxyphenyl) chroman-4-one	42.36	0.21
MOL012245	5,7,4′-Trihydroxy-6-methoxyflavanone	36.63	0.27
MOL012246	5,7,4′-Trihydroxy-8-methoxyflavanone	74.24	0.26
MOL012248	5-Hydroxy-7,8-dimethoxy-2-(4-methoxyphenyl) chromone	65.82	0.33
MOL012250	7-Hydroxy-5,8-dimethoxy-2-phenyl-chromone	43.72	0.25
MOL012251	Chrysin-5-methylether	37.27	0.2
MOL012252	9,19-Cyclolanost-24-en-3-ol	38.69	0.78
MOL002776	Baicalein	40.12	0.75
MOL012254	Campesterol	37.58	0.71
MOL000953	CLR	37.87	0.68
MOL000358	Beta-sitosterol	36.91	0.75
MOL012266	Rivularin	37.94	0.37
MOL001973	Sitosteryl acetate	40.39	0.85
MOL012269	Stigmasta-5,22-dien-3-ol-acetate	46.44	0.86
MOL012270	Stigmastan-3,5,22-triene	45.03	0.71
MOL000449	Stigmasterol	43.83	0.76
MOL000173	Wogonin	30.68	0.23
MOL001735	Dinatin	30.97	0.27
MOL001755	24-Ethylcholest-4-en-3-one	36.08	0.76
MOL002714	Baicalein	33.52	0.21
MOL002719	6-Hydroxynaringenin	33.23	0.24
MOL002915	Salvigenin	49.07	0.33
MOL000351	Rhamnazin	47.14	0.34
MOL000359	Sitosterol	36.91	0.75
MOL005190	Eriodictyol	71.79	0.24
MOL005869	Daucostero_qt	36.91	0.75
MOL000006	Luteolin	36.16	0.25
MOL008206	Moslosooflavone	44.09	0.25
MOL000098	Quercetin	46.43	0.28

**Table 2:** The signaling pathway of KEGG enrichment target number ≥10.

ID	Pathway	Genes	Number of genes
hsa05167	Kaposi sarcoma-associated herpesvirus infection	GSK3B/CASP9/CASP3/CASP8/RELA/CCND1/IL6/VEGFA/FOS/HIF1A/CYCS/RB1/NFKBIA/ICAM1/RAF1/MYC	16
hsa05163	Human cytomegalovirus infection	GSK3B/CASP9/CASP3/CASP8/PRKCA/RELA/CCND1/IL6/VEGFA/CYCS/EGFR/RB1/NFKBIA/MDM2/RAF1/MYC	16
hsa05161	Hepatitis B	BCL2/CASP9/CASP3/CASP8/PRKCA/RELA/IL6/FOS/CYCS/RB1/NFKBIA/PCNA/BIRC5/RAF1/MYC	15
hsa05206	MicroRNAs in cancer	BCL2/CASP3/PRKCA/PLAU/CCND1/TP63/MCL1/VEGFA/EGFR/MDM2/ERBB2/RAF1/MYC/CYP1B1/RASSF1	15
hsa04151	PI3K-Akt signaling pathway	GSK3B/BCL2/CASP9/PRKCA/RELA/CCND1/IL6/MCL1/VEGFA/IGF2/EGFR/MDM2/ERBB2/RAF1/MYC	15
hsa04210	Apoptosis	BCL2/CASP9/CASP3/CASP8/RELA/MCL1/FOS/CYCS/NFKBIA/CASP7/BIRC5/RAF1/PARP1/CTSD	14
hsa05205	Proteoglycans in cancer	ESR1/CASP3/PRKCA/PLAU/CCND1/VEGFA/HIF1A/IGF2/EGFR/MDM2/ERBB2/RAF1/CAV1/MYC	14
hsa05215	Prostate cancer	GSK3B/BCL2/CASP9/PLAU/RELA/CCND1/EGFR/RB1/NFKBIA/MDM2/ERBB2/GSTP1/RAF1	13
hsa05169	Epstein-Barr virus infection	BCL2/CASP9/CASP3/CASP8/RELA/CCND1/IL6/CYCS/RB1/NFKBIA/MDM2/ICAM1/MYC	13
hsa05160	Hepatitis C	GSK3B/CASP9/CASP3/CASP8/RELA/CCND1/CYCS/EGFR/RB1/NFKBIA/RAF1/MYC	12
hsa05170	Human immunodeficiency virus 1 infection	CHEK1/BCL2/CASP9/CASP3/CASP8/PRKCA/RELA/FOS/CCNB1/CYCS/NFKBIA/RAF1	12
hsa04010	MAPK signaling pathway	CASP3/PRKCA/RELA/VEGFA/FOS/IGF2/EGFR/ERBB2/RAF1/MYC/HSPB1/RASA1	12
hsa04115	p53 signaling pathway	CHEK1/BCL2/CASP9/CASP3/CASP8/CCND1/CCNB1/CYCS/MDM2/CHEK2/IGFBP3	11
hsa05210	Colorectal cancer	GSK3B/BCL2/CASP9/CASP3/CCND1/FOS/CYCS/EGFR/BIRC5/RAF1/MYC	11
hsa05162	Measles	GSK3B/BCL2/CASP9/CASP3/CASP8/RELA/CCND1/IL6/FOS/CYCS/NFKBIA	11
hsa04218	Cellular senescence	CHEK1/RELA/CCND1/IL6/CCNB1/RB1/MDM2/RAF1/MYC/CHEK2/IGFBP3	11
hsa05225	Hepatocellular carcinoma	GSK3B/PRKCA/CCND1/IGF2/NQO1/EGFR/RB1/GSTP1/RAF1/MYC/GSTM1	11
hsa05166	Human T cell leukemia virus 1 infection	CHEK1/RELA/CCND1/IL6/FOS/FOSL1/RB1/NFKBIA/ICAM1/MYC/CHEK2	11
hsa05132	Salmonella infection	BCL2/CASP3/CASP8/RELA/IL6/FOS/CYCS/NFKBIA/CASP7/RAF1/MYC	11
hsa05165	Human papillomavirus infection	GSK3B/CASP3/CASP8/RELA/CCND1/VEGFA/EGFR/RB1/MDM2/RAF1/IRF1	11
hsa05022	Pathways of neurodegeneration, multiple diseases	GSK3B/BCL2/CASP9/CASP3/CASP8/PRKCA/RELA/IL6/CYCS/CASP7/RAF1	11
hsa01524	Platinum drug resistance	BCL2/CASP9/CASP3/CASP8/CYCS/MDM2/ERBB2/BIRC5/GSTP1/GSTM1	10
hsa05224	Breast cancer	ESR1/PGR/GSK3B/CCND1/FOS/EGFR/RB1/ERBB2/RAF1/MYC	10
hsa05164	Influenza A	CASP9/CASP3/CASP8/PRKCA/RELA/IL6/CYCS/NFKBIA/ICAM1/RAF1	10
hsa05167	Kaposi sarcoma-associated herpesvirus infection	GSK3B/CASP9/CASP3/CASP8/RELA/CCND1/IL6/VEGFA/FOS/HIF1A/CYCS/RB1/NFKBIA/ICAM1/RAF1/MYC	16
hsa05163	Human cytomegalovirus infection	GSK3B/CASP9/CASP3/CASP8/PRKCA/RELA/CCND1/IL6/VEGFA/CYCS/EGFR/RB1/NFKBIA/MDM2/RAF1/MYC	16
hsa05161	Hepatitis B	BCL2/CASP9/CASP3/CASP8/PRKCA/RELA/IL6/FOS/CYCS/RB1/NFKBIA/PCNA/BIRC5/RAF1/MYC	15
hsa05206	MicroRNAs in cancer	BCL2/CASP3/PRKCA/PLAU/CCND1/TP63/MCL1/VEGFA/EGFR/MDM2/ERBB2/RAF1/MYC/CYP1B1/RASSF1	15
hsa04151	PI3K-Akt signaling pathway	GSK3B/BCL2/CASP9/PRKCA/RELA/CCND1/IL6/MCL1/VEGFA/IGF2/EGFR/MDM2/ERBB2/RAF1/MYC	15

## Data Availability

*S. barbata* was searched in the TCMSP database by entering “Banzhilian”. (https://old.tcmsp-e.com/index.php.). The active ingredients and action targets of *S. barbata* were obtained by screening oral bioavailability (OB) ≥ 30% and drug-like (DL) ≥ 0.18 substances. The keyword “nasopharyngeal carcinoma” was searched through the GeneCards database (https://www.genecards.org/) to obtain the disease target of NPC. The disease target was intersected with the drug target by R software (https://www.r-project.org/), and a Venn diagram was drawn. The PPI network was constructed with STRING plug (https://string-db.org/).
